# Transactions of the Illinois State Medical Society, at Its Tenth Annual Meeting, Held in Paris, May 8th and 9th, 1860

**Published:** 1860-12

**Authors:** 


### BOOK AND PAMPHLET NOTICES.

Transactions of the Illinois State Medical Society, *at its Tenth Annual Meeting, held in Paris, May* 8*th and* 9*th*, 1860. Chicago: Wm. Cravens & Co., 132 Lake-st., Printers.

This is a volume of 226 pages, containing the record of Proceedings of the Annual Meeting, and Reports on Diseases of the Eye, by Dr. E. L. Holmes; on Inflammatory Affections of the Female Breasts, by Dr. W. H. Byford; on Surgery, by Dr. D. Brainard; on the Food most proper for Infants when deprived of the Milk of the Mother, by Dr. N. S. Davis; on the Nature and Treatment of Rheumatism, by Dr. J. S. Whitmire; on the Medical Uses of Veratrum Viride, by Dr. A. Hard; on Practical Medicine, by Dr. C. Goodbrake; on Perineal Pressure to Facilitate Labor, by Dr. T. D. Fitch; and the List of Members who have paid their Annual Assessments. This is the largest and most interesting volume of Transactions ever published by this Society. Copies can be obtained by applying to the Secretary, Dr. N. S. Davis, of Chicago, and enclosing a three cent postage stamp to pay the postage.

---

Transactions of the Indiana State Medical Society, *at its Eleventh Annual Session, held in Indianapolis, May* 17*th and* 18*th*, 1860

